# Technical Advancements for Studying Immune Regulation of Disseminated Dormant Cancer Cells

**DOI:** 10.3389/fonc.2020.594514

**Published:** 2020-11-04

**Authors:** Luigi Ombrato, Marco Montagner

**Affiliations:** ^1^ Barts Cancer Institute, Queen Mary University of London, London, United Kingdom; ^2^ Department of Molecular Medicine, School of Medicine and Surgery, University of Padua, Padua, Italy

**Keywords:** dormancy, metastasis, tumor microenviroment (TME), immune cells, labeling techniques

## Abstract

Metastases are a major cause of cancer-related death and despite the fact that they have been focus of intense research over the last two decades, effective therapies for patients with distant secondary lesions are still very limited. In addition, in some tumor types metastases can grow years after the patients have been declared clinically cured, indicating that disseminated cancer cells (DCCs) persist undetected for years, even decades in a quiescent state. Clinical and experimental data highlight the importance of the immune system in shaping the fitness and behaviour of DCCs. Here, we review mechanisms of survival, quiescence and outgrowth of DCCs with a special focus on immune-regulation and we highlight the latest cutting-edge techniques for modelling the biology of DCCs *in vitro* and for studying the metastatic niche *in vivo*. We believe that a wide dissemination of those techniques will boost scientific findings towards new therapies to defeat metastatic relapses in cancer patients.

## Clinical Problem

According to a recent analysis, the proportion of cancer deaths with metastases as contributing cause, ranged from 9.3% for CNS cancers to 90.4% and 80.2% for ovarian and colon cancer, respectively (1, 2). Metastases can be detected in concomitance with the primary tumor (synchronous) or at a later stage (metachronous). Although most tumors cover the same steps of metastatic dissemination (i.e., extravasation, dissemination through blood or lymphatics, intravazation, and establishment in the metastatic niche), the time required to form overt lesions significantly differs according to the tissue of origin and cancer subtypes. While breast, prostate, renal cell cancers, as well as sarcomas and melanomas show long latency and the time required to develop metachronous metastasis might reach 15 years, 85% of relapses from colon cancer are detected within 3 years (medium latency), and lung cancers often spread at distant sites within a few weeks (short latency) (1, 3–5). When the time required for a DCC to form an overt metastasis after the removal of the primary tumor is long (arbitrarily usually set as 5 years), latency is often referred to as “dormancy”. Importantly, different metastatic latencies might underlie different mechanisms in the acquisition of aggressive traits, and at the same time significantly impact on our capacity to intervene, as the time preceding the metastatic onset offers a therapeutic window so far underexploited. Thus, it is a priority to understand the biology of DCCs, cell intrinsic and extrinsic determinants of their death, survival and growth at the secondary site.

One factor that profoundly affects relapse of DCCs is the cell of origin and its genetic landscape, as exemplified by breast cancers. More than half of breast tumors positive for estrogen receptor (ER) relapse after 5 years of diagnosis and mastectomy, with a progressive increase in recurrence risk from 5 to 20 years in patients treated with adjuvant endocrine therapy (6, 7). This is in contrast with data from patients with ER negative breast cancers, where relapses mostly occur within the first two years (7). Interestingly, while averaging over a long time is required for meta-analysis of different case series, analysing events at shorter intervals in homogeneous case series allows the identification of a multi-peaks pattern of breast cancer recurrence (8, 9). This observation leads to a fundamental question: why do dormant DCCs (DDCCs) reawaken in cured patients with no apparent clinical condition? Beyond stochastic local perturbations, paraphysiological signals involved in exit from dormancy have yet to be identified, but candidates are, for example, hormones or factors related to lifestyle, such as diet (10, 11).

Interestingly, a recent report provided experimental evidence in support of a longstanding clinical observation, i.e., the effect of systemic inflammation on reawakening of DDCCs (8, 12). The paramount role of inflammation and immune surveillance on the behaviour of DDCCs has been unequivocally demonstrated by inadvertently transplanted malignant tumors (13–16). Demand for transplant organs far exceeds available donors, thus, occasionally, donors with a history of cancer were accepted provided that they were disease-free long enough to be considered cured (>10 years). In several cases, recipient patients developed metastases after transplantation of heart, kidney, lungs or liver. Most frequently transplanted tumors were renal cell cancer, cutaneous malignant melanoma, malignant glioblastoma (which is usually considered non-metastatic). Several concepts can be drawn from these reports: i) the presence of a malignant cancer was unknown for some donors, supporting the concept of an early dissemination, ii) the presence of DDCCs in organs that are not considered sites of secondary tumors, indicating that dissemination is not a prerogative of few organs, iii) immune system has a central role in controlling outgrowth of DDCCs, as when the organ was removed and immunosuppression discontinued, malignant cells were rejected by the host (host versus graft). This is supported by the empirical evidence that more metastatic lesions are observed in immunocompromized experimental mouse models compared to wild-type strains (12). The importance of the immune control of DDCCs is further reinforced by the clinical evidence showing discrete peaks of recurrence in patients after resection of the primary tumor, likely as a consequence of systemic inflammation (2, 12, 17). Importantly, perioperative resolution of the inflammatory status prevents outgrowth of otherwise DDCCs (12, 17).

These clinical evidences highlight the role of immunity in the control of DCCs survival and growth and strongly support a better understanding of the dynamic and complex immune tumor microenvironment (TME) at the metastatic site at a single cell level. However, this has been difficult to achieve so far due to the lack of tools to study local interactions between DCCs and their neighbouring cells. In this review we will first clarify key definitions in the dormancy phenotype and then summarise the current knowledge on the non-immune as well as immune-related mechanisms of dormancy. At the end of the review we will highlight recent technological advances that might greatly push forward our knowledge of the molecular mechanisms associated with dormancy.

## Definitions

Dormancy can be used to describe two very distinct phenomena: primary tumor dormancy and metastatic dormancy. The former indicates the time required by an evolving cancer cell to overcome oncogene-induced senescence or apoptosis, metabolic adaptation, evade immune clearance and induce neoangiogenesis, thus forming a detectable tumor mass (18). Metastatic dormancy, instead, indicates the time required by a DCC to overtake the attrition due to seeding in a hostile environment and develop an overt lesion. Although in some cases, determinants of dormancy might be shared among primary tumor and metastases [such as ERK/p38 ratio and fibronectin (19, 20)], they are likely to be distinct processes.

Another distinction often used is between “cellular dormancy”, i.e., cells undergoing reversible G0/G1 cell cycle arrest, and “tumor mass dormancy”, indicating small clusters of cells where proliferation is balanced by death induced by lack of nutrients (angiogenic dormancy) and/or by immune clearance (immune dormancy). Although useful to rationalize the dormant phenotype, this sharp distinction has little experimental support and likely the two conditions coexist, with DDCCs dynamically fluctuating between the two states during their history. For example, a small, but detectable, proportion of early DCCs (eDCCs) from experimental breast cancers are found positive for proliferation markers (21), despite they are often referred to as “non-proliferative”. Moreover, Aguirre-Ghiso and colleagues showed, with an elegant genetically-encoded fluorescent marker dilution assay, that post-hypoxic DCCs, which are much less proliferative than post-normoxic DCCs, still undergo considerable proliferation over two weeks (22). This dynamic heterogeneity is not unique to DDCCs, as a significant number of Ki67-negative cells are found even in DCCs from aggressive cell lines, such as MDA-MB-231 (23). The development of longitudinal assays that keep track of the proliferative history of DCCs will help to understand if cellular and tumor mass dormancy are static or dynamic entities and which of them contributes to aggressive lesions.

## Mechanisms of Survival, Quiescence, and Reawakening of DDCCs

The fate of disseminated cells is driven by a combination of cell intrinsic, extrinsic and stochastic events (1). Cell intrinsic programs involve oncogenes and tumor suppressors, membrane proteins (integrins, receptors etc…), intracellular components (such as cytoskeletal proteins and mechanotransducers), signaling pathways and sensors that integrate genetic and microenvironmental inputs and translate them into cellular processes. Cell extrinsic programs include triggers from the niche, such as stromal cells, tissue architecture, biophysical and biochemical cues, as reviewed in (24, 25). Intrinsic and extrinsic signals do not act on their own, rather they are nodes and connectors of a complex and dynamic network where extrinsic signals from TME (organ specific or shared) funnel into key intrinsic signaling hubs.

### Immune-Independent Mechanisms

P-ERK/P-p38 ratio is perhaps the most widely validated dormancy hub so far (25, 26). While activated ERK drives exit from dormancy and growth, P-p38 promotes growth arrest *via* several mechanisms, such as MSK1, DEC2, NDRG1, NR2F1, and ultimately p21 and p27 induction (26, 27). Several signals converge on p38, such as TGFβ2 (28), BMP7 (29) as well as the metastasis suppressors MKK4 and Nm23 (30–32). Another determinant of dormancy/growth signaling is the PI3K/Akt/mTOR axis, whose activation drives survival and exit from dormancy (33–37). Different integrin dimers, often in conjunction with Src, have been consistently linked with survival of DDCCs and/or metastatic outbreak (20, 24, 38–45). Several signaling pathways have also been linked so far with quiescence and metastatic fitness: TGFβ and BMP pathways (23, 28, 29, 46–48), canonical and non-canonical Wnt pathway (21, 49–51), YAP/TAZ (41, 42), Notch (49, 52), JAK/STAT (53, 54). Recently, ER stress response and autophagy have been convincingly linked with survival of DDCCs *in vitro* and *in vivo* (55–58).

Several fibrous and non-fibrous ECM proteins have been shown to be key determinants of metastatic fitness (40): collagen I (39, 59), fibronectin (20, 38, 60), periostin (23, 50), tenascin C (49), thrombospondin (23, 41). Beside ECM proteins, hypoxia present at the primary site primes breast cancer DCCs for dormancy upon seeding to secondary organs (22).

Stromal cells provide organ-specific niches that regulate both quiescence and reactivation. Bone is probably the most characterised niche, since it is the preferred metastatic target of prostate and breast cancer (61). Osteoblasts release the growth arrest specific 6 (GAS6) ligand that binds to the Axl subfamily of receptors inducing dormancy (47, 62–65). Importantly, DDCCs can hijack endogenous signals regulating hematopoietic stem cells’ (HSC) reversible quiescence. For example, the chemokine CXCL12 from bone endothelial cells and mesenchymal progenitors induces dormancy in DCCs and HSC (1, 66–69). On the contrary, in preclinical models of bone metastasis, RANK-stimulated osteoclasts are reported to mobilize DDCCs and trigger proliferation (43, 70–72). Lung is another common homing site for DDCCs and interaction of breast DDCCs with type I pneumocytes is key for the activation of a dormant gene program in DCCs (20). In this context, cellular protrusions are required to gather survival signals from the microenvironment (20, 73, 74). Importantly, stromal derived BMP2 and TGFβ2 keep DDCCs in a latent state (28, 46), while collagen-rich fibrotic lung transforms DDCCs into aggressive metastatic cells (39), a similar mechanism was observed in fibrotic liver (75). Lastly, the perivascular niche regulates DCCs behaviour and chemoresistance in multiple organs (23, 45, 76).

### Immune-Related Mechanisms

Immune cells are known to play a key role in shaping the TME in primary tumor and metastasis (77–79) and several evidences show that their recruitment at distant sites anticipates cancer cells colonization (59, 80–86). Moreover, extracellular vesicles (EVs) from the primary tumor have been shown to induce a premetastatic niche at the metastatic site [reviewed in (87, 88)]. Notably, the protein content of exosomes is critical to their function and it defines where cancer cells metastasise (89) and also influences response to chemotherapy of DCCs (90).

Because of their acknowledged tumor modulatory function immune cells have not surprisingly become a valid therapeutic target. Immunotherapy has finally proven its efficacy in treating patients and promises to further change the standard of care for cancer treatment in the coming years (91–94). However, a complete resolution of the TME as well as the understanding of this local crosstalk is far to be achieved, possibly limiting the efficacy of current immunotherapeutic options to a small number of patients. This local crosstalk has been shown to also occur *via* EVs. Immune cell derived exosomes have been initially shown to function as immunomodulators by carrying molecules able to induce a T-cell response (95, 96). However, metastatic cells can release exosomes expressing PD-L1 on their surface and are therefore able to suppress cytotoxic T cells (97–99). These mechanisms might influence a positive response to immunotherapy. Moreover, a further boost in immunotherapy might come from a better understanding of the immune diversity in the TME and the way immune cells locally interact, as this can help to predict therapeutic responsiveness (91, 100). Since immune cells are important in limiting metastatic outgrowth and keeping DCCs in an indolent state (5, 26, 101, 102), it is tempting to foresee a role of immunotherapy in targeting dormant DCCs (103). However, this possibility is currently restrained by a limited understanding of how immune cells interact with DCCs. In the next sections we will summarise the current knowledge on the role of immune cells specifically in metastatic dormancy.

#### Innate Immunity and Dormancy

Macrophages have long been known to play a role in cancer (77) and the intriguing finding that they polarise their status to support cancer growth paved the way for studies on immune cell pro-tumorigenic functions. For example, macrophages support tumor growth by several means, among them a direct inhibition of tumor suppressive immune cells (104, 105). Macrophages are able to directly promote breast DCCs survival in the lung *via* a VCAM1-α4 integrin binding (44). Interestingly, the aberrant expression of VCAM1 in bone-disseminated breast DCCs promotes the recruitment of monocytic osteocytic progenitors and subsequent transition from indolent growth to overt bone metastasis (43). Macrophages also sustain early dissemination and metastasis in the HER2+ model of breast cancer (106) and have been shown to interact with residual tumor cells and promote tumor recurrence in a HER2-driven breast cancer (107).

Neutrophils represent another abundant component of innate immunity whose contribution in cancer has only started to be elucidated in the last few years (108). Neutrophils have been shown to boost lung metastasis from breast cancer (81, 109–111) and to reawaken DDCCs (41, 107). Their ability to reawaken DDCCs in the lung following LPS exposure is strongly dependent on the release of neutrophils extracellular traps (NETs) (41). Notably, the metastatic outgrowth of DDCCs induced by LPS-mediated inflammation is rescued following neutrophil depletion, but not when depleting macrophages with anti-CCL2 (107), indicating a unique role for neutrophils in this context.

Another innate immune population, the NK cells, has been associated with the clearance of DCCs. NK cells play a key role in immune surveillance during metastatic dissemination (112). Indeed, the expression of NK cell-activating ligands on cancer cells is critical for their clearance (113) and the upregulation of NK cell-activating receptors render cancer cells more susceptible to NK cell-mediated killing (114). Moreover, neutrophils-mediated NK-cell depletion promotes outgrowth of disseminated carcinoma cells (115). By using a “latency competent model” of breast and lung carcinoma, Massague and colleagues showed that while NK cells clear most of the disseminated cells at first instance, some cancer cells stochastically enter quiescence and downregulate ligands for NK cells to evade the immune surveillance. Importantly, these quiescent DCCs keep their tumorigenic potential and can re-enter cell cycle to metastases when NK surveillance is released (116).

#### Adaptive Immune System and Dormancy

Cancer immunoediting has been recognized as a process by which the immune system controls cancer growth, with a primary role of adaptive immunity (117, 118). Schreiber and Smyth laboratories made important contributions to show how T cells maintain cancer cell quiescence and how the depletion of CD4+ and CD8+ cells, but not NK cells, allows primary tumor growth in a carcinogen-induced model of sarcoma (119, 120). A key role for a subpopulation of T cells, the tissue-resident memory CD8+ cells, in maintaining a durable immune-cancer equilibrium, has also been shown in skin melanoma (121).

Importantly, T-cells also control cancer outgrowth when DCCs colonize secondary sites. Persistent endoplasmic reticulum (ER) stress plays a role in maintaining pancreatic DCCs quiescence and protecting them from a CD8+ T-cell-mediated response. Indeed, the combination of ER stress relief and T-cell depletion allows liver metastasis formation (57). Another study supports a pre-eminent role for CD8+ T-cells, but not CD4+ cells, in the immunosurveillance of DCCs in a model of spontaneous melanoma (122). CD8+ T-cells have also been shown to induce a state of dormancy in murine B cell lymphoma *via* the production of INFy (123), while in fibrosarcoma, either CD8+ T-cell or NK cell depletion lead to spontaneous metastasis in immune-competent mice. An orchestrated response involving different immune subpopulations has been suggested in a model of chemotherapy-induced dormancy in ER negative breast cancer. Here, the signaling activation of the IRF7/IFN-β/IFNAR axis following chemotherapy is associated with a reduction in granulocytes and expansion of T and B lymphocytes and dendritic cells (124).

## *In Vivo* Mouse Models to Study Immune-DDCCs Crosstalk

In the past decades several mouse models have been generated, each of them with strength and limitations in modeling the metastatic cascade [reviewed in (125)]. Experimental metastatic spread can be achieved i) by spontaneous dissemination of cells after formation of a primary tumor (either by cancer cell transplant or genetically-induced) or ii) by injecting cancer cells in the bloodstream (either allografts of mouse cells, or xenografts of human cells). The former have the advantage to mimic all the stages of metastatic colonization, the latter are more rapid and allow genetic manipulation of the injected cells, a prerequisite for some labeling techniques described later. Transgenic mouse models of dormant/indolent metastatic mammary cancers are worth mentioning, because they provide a good opportunity to study DDCCs in an *in vivo* immunocompetent animal. So far, three transgenic breast cancer models with spreading of indolent BCCs (breast cancer cells)have been reported: MMTV-Her2 (126), MMTV-PyMT (127), MMTV-Wnt1 (128). MMTV-Her2 and MMTV-PyMT models were used to support the early dissemination hypothesis, whereby dormant BCCs could be retrieved from the lung before the detection of the primary tumor lesion (127). Similarly, mammary cancers originating from the MMTV-Wnt1 transgene spread asymptomatic cells to lung and lymph nodes (128). Importantly, disseminated cells can be reawakened from dormancy following systemic triggers like bone marrow transplant or surgery. More recently, MMTV-Her2 and MMTV-PyMT mice have been used to describe the role of progesterone receptor, Her2 and partial-EMT into early dissemination (21, 126). The main limitation of the aforementioned models is that murine and human immune systems have important differences that undermined clinical translation of several preclinical findings. Moreover, the use of human cancer cells requires the use of immunodecifient or immunocompromised mice, which obviously fail to capture DDCCs-immune TME crosstalk.

Development of “humanized mice” started thirty years ago with the aim of studying human diseases in mice with a human immune system. Humanized mice are immunocompromised mice transplanted with human peripheral blood mononuclear cells (PBMCs), hematopoietic stem cells (HSCs) or human fetal tissues (thymus and liver) (129). Engraftment of patient-derived xenograft, or PDX (tumor fragments or single-cell suspension from tumor resections), are also employed to reconstitute the TME in mouse models. So far these models have been exploited mainly as preclinical testing platforms, as treatment responses in PDX have been correlated to those observed in patients in several cancer types (130), but they hold great potential to uncover previously overlooked human-specific aspects of immune-DDCCs crosstalk.

## Technological Advances in Studying the Tumor Microenvironment

**Figure 1:**
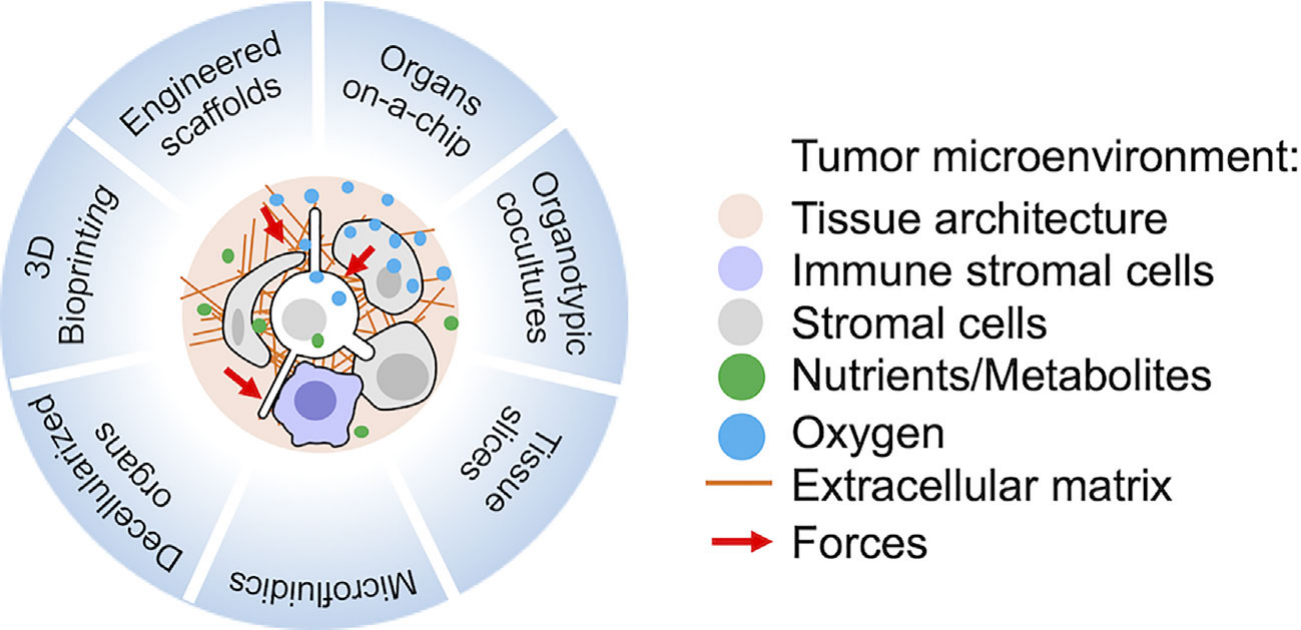
Recent technologies suitable for the study of metastatic tumor microenvironment (TME) *in vitro* or *ex vivo*. Relevant reviews with references to the original works and protocols are provided in the main text.

Advances in biomaterial technologies, including 3D bioprinting, are fundamental to model TME *in vitro* (131–136). The use of complex multi-cultures to mimic and perturb metastatic dormancy *in vitro* has been rapidly expanding, as reviewed in (24). More recently, the advances in microfluidic technologies are also boosting the development of cancer-on-chip models to better recapitulate multiple parameters of the TME complexity *in vitro* (137, 138) (**Figure 1**).

### Biomaterial Technologies and 3D Bioprinting

The availability of new biomaterials also improved the studies of metastatic and immune cell interactions *in vivo*. Advancements in biomaterials allow to mimic the natural architecture of human tissues with scaffolds of tunable properties, either of natural (for example Matrigel) or synthetic origin (such as PCL, PLGA or PEG, polycaprolactone, polylactic-co-glycolic acid, polyethylene glycol, respectively) (132, 134). 3D scaffolds have been used to study the effects of ECM components and physical tissue properties as well as to dissect interactions between disseminated cells and stromal cells, such as fibroblasts, endothelial cells and macrophages (24, 134). Heterotopically implanted 3D hydrogels have been used to recreate artificial metastatic niches *in vivo*. Interestingly, following implantation they were infiltrated by immune cells and able to attract DCCs (139–141). Moreover, these systems can be easily manipulated to release cytokines and attract specific immune populations (140), representing a powerful tool to study how metastatic cells interact with host cells *in vivo* at a molecular level. However, they do not reflect the actual metastatic site composition whose replication remains difficult. This challenge has been partially overcome for leukemic tumors, thanks to the ability of HSC and progenitor cells to engraft and re-create a bone marrow environment. Humanized bone marrow environments have been used to study cellular interactions with human HSCs as well as malignant leukemic cells (142, 143). Interestingly, the engineered human bone-marrow niche can recapitulate main features of the pre-metastatic niche and attract DCCs, allowing to study the progression of the metastatic cascade (144). Importantly, 3D scaffolds have been successfully used as platforms for drug screening.

Three-dimensional bioprinting represents the most sophisticated strategy to achieve spatial control of matrix properties, integration of perfusable vascular networks and precise cancer-stroma cellular interaction (135). With 3D bioprinting, tissue spheroids, microcarriers, cell clusters, pellets, biomaterials and/or decellularized ECM can be deposited as bioinks under the control of computer designed patterns (135). With this technology, cancer models for several tissues have been generated, suggesting that metastatic niches as well could be designed in the near future.

### Decellularized Organs and Precision Cut Tissue Slices

Hundreds of ECM proteins and carbohydrates are known to date, and their combination is key to the specificity of any cellular niche. Thus, reconstituting the exact ECM composition *in vitro* is almost impossible. For these reasons, several groups developed protocols to remove the cellular components of cell/ECM constructs, leaving decellularized ECM (dECM) that can be used for advanced *in vitro* model systems. dECM can be derived from native tissues or from tissues/organs generated *in vitro*. Decellularization protocols include chemical, physical or enzymatical approaches (or combinations) and the method clearly affects how much the resulting dECM resembles the native ECM of origin (145, 146). Decellularized tissues have been used to mimic breast cancer colonization of lungs (147) and adipose tissue (148). Importantly, dECM can be derived from patients, this allowed Pinto and colleagues to study crosstalk between colon cancer cells and macrophages within dECM from healthy and compromised tissues (149).

Long term *ex vivo* cultures of precision cut tissue slices and decellularized organs could be repurposed to study TME-DDCCs crosstalk in several secondary organs, such as lung, liver and brain (146, 150–153). Moreover, the use of intravital imaging technology combined with skin-fold chambers or optical windows has also allowed researchers to examine complex events *in vivo*, particularly in the context of primary tumors (154–156), but also in studying metastasis in organs such as bone, brain, liver and lung (157–159). However, the study of the metastatic TME *in vivo* remains technically difficult, particularly at an early stage of the disease, when small tumor nodules need to be spatially located in the metastatic tissue. This challenge is even bigger when dormant cell clusters or single DCCs need to be visualised and their neighbouring cells identified.

### Microfluidic Systems and Organs-on-a-Chip

Another significant technological improvement for the design of metastatic niche *in vitro* is the development of microfluidic scaffolds. These platforms allow modelling of barriers and interfaces of tissues as well as a tight control of forces, perfusion and strains. Interfaces can be based on synthetic materials, hydrogels or self-assembled (160, 161). Organs-on-a-chip employ a combination of all the above techniques to generate organotypic models with geometrically defined multicellular composition, mechanical/electrical/biochemical stimulation and controlled liquid flow (160, 161). Organs-on-a-chip have been generated for lungs, heart, kidney, liver, muscle, while chips recapitulating immune responses have been underexplored so far, with the notable exceptions reviewed in ref. (162).

### Laser-Capture Microdissection

The introduction of laser-capture microdissection technology has been largely used to study TME over the last 15 years (163, 164). The possibility to laser-cut a piece of tumor from a tissue section and specifically isolate cells from the TME by fluorescence activated cell sorting (FACS) enormously contributed to our knowledge in the field. However, despite being a powerful methodology this has some major limitations, mostly due the quality of the isolated material from a fixed tissue. Moreover, this approach could be very complicated to adopt when the spatial location of small metastatic nodules is required. Techniques involving the labeling of stromal cells within the niche could overcome these limitations. Once labeled, these cells can be isolated as live cells by flow cytometry, allowing their functional characterization *ex vivo*. Nowadays, the possibility to couple *in vivo* labeling techniques with state-of-art single cell analysis could enormously extend what we know about the role of the TME in the coming years.

### *In Vivo* Labeling of Metastatic Niche

In this paragraph, we will discuss in detail some recently developed *in vivo* labeling methods. These systems have potential to be optimized in the context of dormancy and may finally reveal the “dormant niche” *in vivo*.

The techniques most commonly used to identify and isolate cells from tissues, including the most recent ones we describe in this section, imply using Fluorescence-Activated Cell Sorting (FACS) during the procedure. FACS has indeed proved to be a key asset to study the TME and the use of specific cellular markers has been critical to characterise different cell populations within the TME. In **Supplementary Table 1**, we provide a list of markers that might be useful to identify the cellular populations in the TME (this list has to be considered as a simplified guide to roughly discriminate the most abundant cellular components and need to be refined according to specific experimental needs and continuously revised as new findings emerge). However, advances in new technologies, and particularly the advent of the single cell RNA sequencing (scRNA-seq), keep revealing how the expression of markers initially thought to be exclusive of one lineage population are actually shared among different cell populations. Moreover, high heterogeneity and plasticity have been observed within the same cellular components in the TME, for example in tumor-associated macrophages and cancer-associated fibroblasts among the others (165–170). All this complexity makes it difficult to distinctively isolate some cellular sub-populations. The possibility to couple unbiased niche labeling methods with scRNA-seq could help to define more precise combinations of markers to identify specific subpopulations.

**Figure 2:**
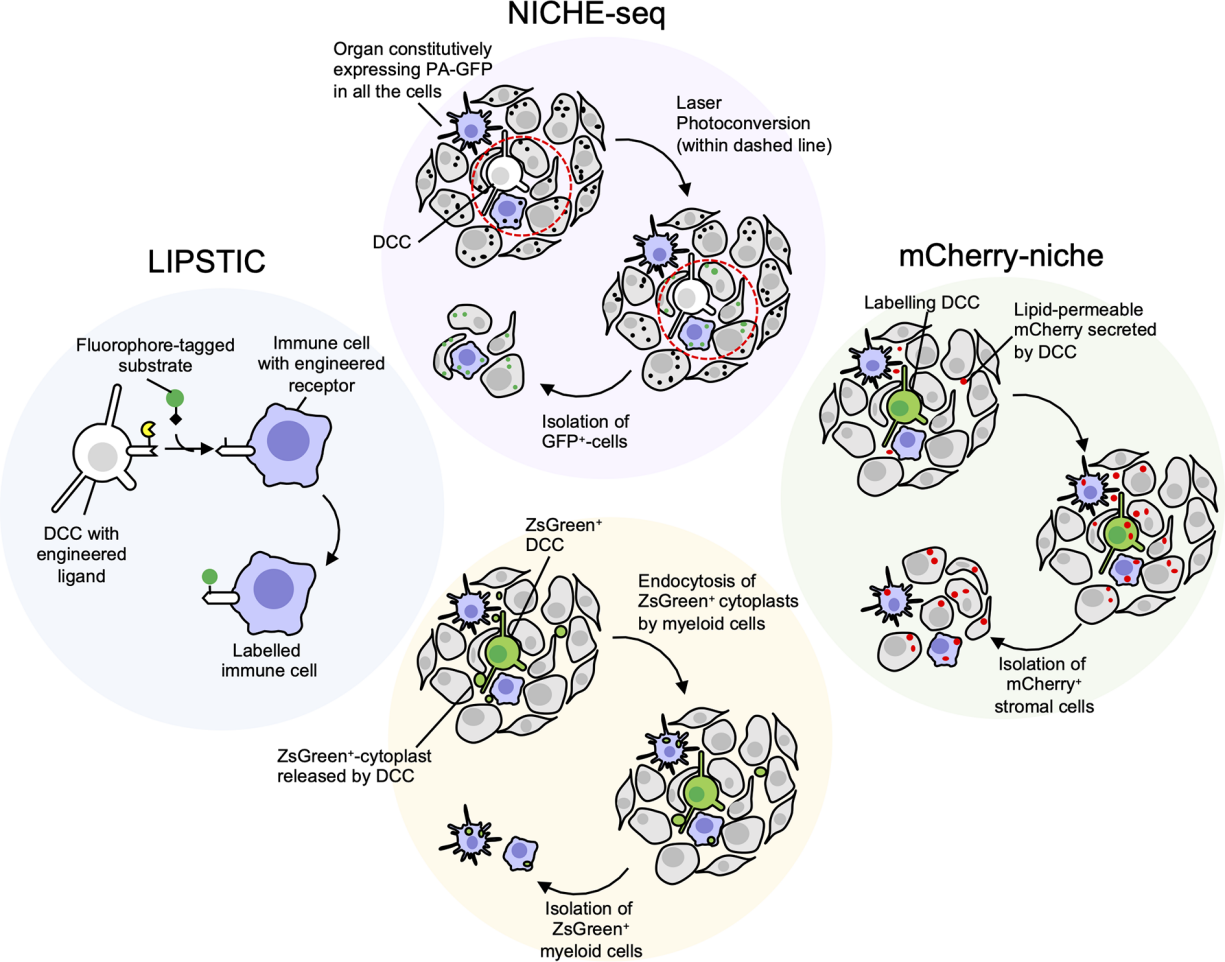
Niche-labeling techniques for characterization of immune metastatic tumor microenvironment (TME). White/Green: cancer cells; Grey: stromal cells; Purple: immune stromal cells. NICHE-seq (172) employs transgenic mice constitutively expressing photoactivatable GFP (PA-GFP), a fluorescent protein that increases its emission after excitation with 413 nm light (black dots: dark state; green dots: fluorescent state after photoconversion). Once a disseminated cancer cell (DCC) is located in the metastatic organ the surrounding niche can be irradiated and GFP^+^ cells isolated. Main limitations: i) the difficulties to spatially locate few scattered dormant DCCs (DDCCs) throughout entire organs; ii) the accessibility of those organs for photoconversion *in vivo*. The latter issue can be overcome with *ex vivo* photoconversion of freshly explanted organs. LIPSTIC (labeling Immune Partnerships by SorTagging Intercellular Contacts) (173) is an intercellular enzymatic labeling technique that exploits *Staphylococcus aureus* transpeptidase sortase A (SrtA, in yellow). Here, SrtA transfers a substrate containing “LPXTG” motif (black diamond), fused with biotin or fluorophore, to five N-terminal glycine residues tag (G5). This transfer requires proximity of SrtA and G5, thus a receptor and its membrane-bound ligand are fused with either SrtA and G5 in different cells. If these cells, that could be DDCCs and stromal cells, lie in close proximity at the metastatic site, stromal cells surrounding DDCCs are labeled and can be isolated for further analysis. Main limitations: i) the stromal lineage of interest must be genetically engineered *a priori* with tagged receptor or ligand, making this technique not suitable for unbiased identification of niche stromal cells; ii) the cells must be in close proximity for the reaction to happen. Cherry-niche (172) was developed to overcome these limitations. Here, the mCherry protein is engineered with a lipid-permeable domain (sLP-mCherry). DCCs expressing the sLP-mCherry release the protein in the extracellular space and the protein is uptaken by neighboring cells that can be isolated and analysed. Similarly, Krummel lab observed that blebs (cytoplasts, green dots) released by disseminated ZsGreen^+^-melanoma cells are endocytosed by resident myeloid cells (159). This approach is limited to DCCs releasing a significant amount of blebs and to stromal cells with efficient endocytic capacity.

The generation of a genetic mouse model expressing a photoactivatable GFP (171) coupled with the two-photon microscopy technology allowed photoactivation of specific regions of inguinal lymph nodes with a technique called NICHE-seq (**Figure 2**) (172). Labeled cells were isolated by FACS and analysed by single cell RNA-sequencing. The same approach has shown potential to photoactivate regions surrounding melanoma cells (172). The main limitation is represented by the physical accessibility of the tissue to imaging and photoactivation, and by the requirement to precisely locate the cancer cell within an entire tissue, that can be particularly challenging in the case of isolated DDCCs.

To overcome this limitation, Headley and colleagues engineered melanoma cells to express cytoplasmic Zs-green, a highly brilliant fluorescent protein, that is also incorporated in tumor cell fragments (cytoplasts). By endocyting these fragments, neighboring cells become fluorescent themselves and can be visualised or isolated (159). The efficacy of this method depends on the amount of microvesicles the tumor cells release and on the ability of the neighbouring cells to internalise/phagocyte them, therefore limiting cell detection mostly to myeloid immune cells.

In an alternative method called LIPSTIC, a receptor-ligand interaction can be marked by the transferring of a biotin-tag on the recipient cells (173). Here, “donor” T cells expressing a CD40L fused to Sortase A interacted with B-cells engineered to express an “acceptor” domain fused to the CD40 receptor. When the receptor-ligand interaction occurs in presence of a fluorescent or a biotinylated substrate, the acceptor cells are labeled. This strategy implies a physical interaction between cells, and a ligand-receptor pair previously engineered and expressed by the right cell lineage(s). Moreover, the ectopic expression of endogenous ligand-receptors may cause unwanted biological effects, thus suggesting the need to engineer more neutral synthetic systems.

Another approach that we have recently developed, named Cherry-niche, allows engineered cancer cells to label their surroundings by transferring a modified red-fluorescent protein (174). Neighbouring cells of the cancer cells endocyte this protein and become fluorescent. Thanks to its liposoluble features, Cherry-niche does not require direct cell-cell contact nor a-priori knowledge of the recipient cells, as all the surrounding cells have the potential to internalise the fluorescent tag. Importantly, in *in vivo* organs, such as in the lung, the bulk of the labeling is limited to the close proximity of the metastatic cells, highlighting the potential of Cherry-niche to specifically reveal the cancer neighbouring cells.

## Conclusions

In the last decade tremendous advancements have been achieved in oncology following the development of cutting-edge techniques. Among the different aspects of cancer biology, survival, quiescence and outgrowth of DCCs remained underexplored due to experimental hurdles such as faithfully modeling of metastatic organs *in vitro* and labeling of metastatic TME *in vivo.* In this review we presented recent techniques that in our opinion will give great impulse towards these directions. Despite this, our knowledge of DDCCs in human patients is extremely limited. This is mostly due to the current lack of techniques to track single or small clusters of DDCCs, together with ethical and technical issues with collecting and analysing metastatic organs in cured healthy patients. A notable exception is the bone marrow, a frequent site of relapse for several cancers. From this tissue, single DDCCs have been isolated, profiled (175) and provided clinical evidence of the existence of DDCCs in patients with no evidence of disease (176, 177). Isolation of circulating tumor cells or circulating tumor-derived factors from blood biopsies holds great potential to bypass the aforementioned limitations (178), although work is still needed to identify DCCs with metastasis-forming ability from a heterogeneous population of DCCs. Moreover, DDCCs do not effectively respond to chemotherapies or radiation therapies as a consequence of quiescence and because of the protective role of microenvironment (45, 179), thus, immunotherapy holds great hopes for clearing organs from DDCCs before relapse. In the light of this, it will be of utmost importance to exploit the most recent techniques to deepen our knowledge of DDCCs-immune cells crosstalk at the metastatic site.

## Author Contributions

LO and MM equally contributed to conceiving and writing the review. All authors contributed to the article and approved the submitted version.

## Funding

LO was funded by a Barts Charity Lectureship (grant MGU045). MM also received funding from Marie Curie Actions-Intra-European Fellowships no. 625496 and BIRD Seed grant from Department of Molecular Medicine (University of Padua).

## Conflict of Interest

The authors declare that the research was conducted in the absence of any commercial or financial relationships that could be construed as a potential conflict of interest.

## Supplementary Material

Supplementary Table 1 Lineage markers to identify cell populations in the murine TME. TME components have been roughly categorised here in broad stromal cell types (immune, endothelial, mesenchymal and parenchymal). CD45 can be used to identify all the immune cells. To further discriminate subpopulation within the immune compartment it needs to be combined with other markers. For example, a combination of CD45, CD11b and Ly6G will distinctively identify the neutrophils. This list represents a very simplified guide to roughly discriminate the most abundant cellular components in the TME, therefore not including all type of cells that might be found. We recommend to refine the marker combination to use according to specific experimental needs. Moreover, new findings may need to be considered to implement or update this list.
