# Supplementary material for: Technical Advancements for Studying Immune Regulation of Disseminated Dormant Cancer Cells
Source: Front Oncol. 2020 Nov 4;10:594514. doi: 10.3389/fonc.2020.594514 (PMC7672194; doi:10.3389/fonc.2020.594514)
Supplement: Supplementary Table 1 — Lineage markers to identify cell populations in the murine TME. TME components have been roughly categorised here in broad stromal cell types (immune, endothelial, mesenchymal and parenchymal). CD45 can be used to identify all the immune cells. To further discriminate subpopulation within the immune compartment it needs to be combined with other markers. For example, a combination of CD45, CD11b and Ly6G will distinctively identify the neutrophils. This list represents a very simplified guide to roughly discriminate the most abundant cellular components in the TME, therefore not including all type of cells that might be found. We recommend to refine the marker combination to use according to specific experimental needs. Moreover, new findings may need to be considered to implement or update this list. [file DataSheet_1.pdf]

TME compartment	Marker			Localisation	Population identified	Notes
**Immune compartments**	CD45			Membrane	Immune cells	All immune cells express CD45 and can be broadly divided in myeloid cells and lymphoid cells due to the presence of either CD11b or CD3 on their membrane. However, exceptions exist as for the NK cells that are still considered lymphoid cells though they are CD3 negative and some express CD11b.
		CD11b		Membrane	Myeloid cells	
			Ly6G	Membrane	Neutrophils	
			CD14	Membrane	Monocytes	
			F4/80	Membrane	Macrophages	
			CD11c	Membrane	Dendritic cells	
		CD3		Membrane	T cells	
			CD4	Membrane	Helper T cells	
			FoxP3	Intracellular	Regulatory T cells	
			CD8	Membrane	Cytotoxic T cells	
		NKp46		Membrane	NK cells	
		CD19		Membrane	B cells	
**Endothelial compartment (CD45$^-$)**	Endomucin			Membrane	Endothelial cells	Endothelial cells are often present in tumour. CD31 is a traditionally that has been largely used to identify them by FACS.
	VE-cadherin			Membrane	Endothelial cells	
	CD31			Membrane	Endothelial cells	
		Lyve-1		Membrane	Lymphatic endothelial cells	
**Mesenchymal compartment (CD45$^-$)**	FAP			Intracellular		Cancer-Associated Fibroblasts (CAFs) represent the most abundant mesenchymal non-immune component in the TME. The origin of CAF heterogeneity remains debated and there is not a single marker able to recognise all CAFs.
	aSMA			Intracellular		
	Vimentin			Intracellular		
	CD29			Membrane		
	Ly6A			Membrane		
	PDGFRb			Membrane		
**Parenchymal compartment (CD45$^-$)**	Epcam			Membrane	Epithelial cells	In most solid tumour the parenchymal cells are mainly represented by the host tissue epithelial cells. Epcam is a membrane marker commonly used to identify epithelial cells. However, there are markers specific for each particular tissue that might be useful to more clearly identify epithelial cells, including local subpopulations that can be Epcam negative.
